# Association between diabetic foot ulcer and diabetic retinopathy

**DOI:** 10.1371/journal.pone.0175270

**Published:** 2017-04-07

**Authors:** Duck Jin Hwang, Kyoung Min Lee, Moon Seok Park, Sung hee Choi, Ji In Park, Joon Hee Cho, Kyu Hyung Park, Se Joon Woo

**Affiliations:** 1 Department of Ophthalmology, Seoul National University Bundang Hospital, Seongnam, Korea; 2 Department of Ophthalmology, HanGil Eye Hospital, Incheon, Korea; 3 Department of Orthopedic Surgery, Seoul National University Bundang Hospital, Seongnam, Korea; 4 Department of Endocrinology, Seoul National University College of Medicine, Seoul National University Bundang Hospital, Seongnam, Korea; 5 Department of Internal Medicine, Kangwon National University Hospital, Chuncheon, Korea; 6 Department of Ophthalmology, Kangnam Sacred Heart Hospital, Hallym University College of Medicine, Seoul, Korea; 7 Department of Ophthalmology, Seoul National University College of Medicine, Seoul, Korea; University of Colorado Denver School of Medicine, UNITED STATES

## Abstract

**Purpose:**

We aimed to investigate the prevalence of diabetic retinopathy (DR) in patients with diabetic foot ulcer (DFU) and elucidate the association between DR and DFU severities and their shared risk factors.

**Methods:**

A retrospective review was conducted on DFU patients who underwent ophthalmic and vascular examinations within 6 months; 100 type 2 diabetic patients with DFU were included. The medical records of 2496 type 2 diabetic patients without DFU served as control data. DR prevalence and severity were assessed in DFU patients. DFU patients were compared with the control group regarding each clinical variable. Additionally, DFU patients were divided into two groups according to DR severity and compared.

**Results:**

Out of 100 DFU patients, 90 patients (90%) had DR and 55 (55%) had proliferative DR (PDR). There was no significant association between DR and DFU severities (R = 0.034, p = 0.734). A multivariable analysis comparing type 2 diabetic patients with and without DFUs showed that the presence of DR [OR, 226.12; 95% confidence interval (CI), 58.07–880.49; p < 0.001] and proliferative DR [OR, 306.27; 95% CI, 64.35–1457.80; p < 0.001), higher HbA1c (%, OR, 1.97, 95% CI, 1.46–2.67; p < 0.001), higher serum creatinine (mg/dL, OR, 1.62, 95% CI, 1.06–2.50; p = 0.027), older age (years, OR, 1.12; 95% CI, 1.06–1.17; p < 0.001), higher pulse pressure (mmHg, OR, 1.03; 95% CI, 1.00–1.06; p = 0.025), lower cholesterol (mg/dL, OR, 0.94; 95% CI, 0.92–0.97; p < 0.001), lower BMI (kg/m^2^, OR, 0.87, 95% CI, 0.75–1.00; p = 0.044) and lower hematocrit (%, OR, 0.80, 95% CI, 0.74–0.87; p < 0.001) were associated with DFUs. In a subgroup analysis of DFU patients, the PDR group had a longer duration of diabetes mellitus, higher serum BUN, and higher serum creatinine than the non-PDR group. In the multivariable analysis, only higher serum creatinine was associated with PDR in DFU patients (OR, 1.37; 95% CI, 1.05–1.78; p = 0.021).

**Conclusions:**

Diabetic retinopathy is prevalent in patients with DFU and about half of DFU patients had PDR. No significant association was found in terms of the severity of these two diabetic complications. To prevent blindness, patients with DFU, and especially those with high serum creatinine, should undergo retinal examinations for timely PDR diagnosis and management.

## Introduction

More than 25 million people in the United States are estimated to have diabetes mellitus (DM), and 15–25% will develop a diabetic foot ulcer (DFU) during their lifetime [1]. DFU is one of the most serious and disabling complications of DM, resulting in significantly elevated morbidity and mortality. Vascular insufficiency and associated neuropathy are important predisposing factors for DFU, and DFU is the most common cause of non-traumatic foot amputation worldwide. Up to 70% of all lower leg amputations are performed on patients with DM, and up to 85% of all amputations are preceded by a DFU [2, 3]. Every year, approximately 2–3% of all diabetic patients develop a foot ulcer, and many require prolonged hospitalization for the treatment of ensuing complications such as infection and gangrene [4, 5].

Meanwhile, a number of studies have noted that diabetic retinopathy (DR) is associated with diabetic neuropathy and microvascular complications [6–10]. Despite the magnitude of the impact of DFUs and their consequences, little research has been performed to investigate the characteristics of patients with a DFU and DR. Moreover, to the best of our knowledge, no prior study has addressed the prevalence of DR in patients with a DFU, and no direct association between the severities of DR and DFU has been reported to date. Considering the pathogenic mechanisms shared between DR and DFU, we hypothesized that there is a relationship in the clinical features between the two diseases.

Therefore, the aim of this study was to investigate the prevalence of DR in patients with a DFU and to elucidate the potential association between DR and DFUs.

## Methods

### Subjects

#### Patients with DFUs

A retrospective review of consecutive patients who visited the Department of Orthopedics at Seoul National University Bundang Hospital between October 2004 and October 2011 was conducted. Inclusion criteria were as follows: (1) all type 2 diabetic patients diagnosed with a DFU based on the Wagner ulcer classification; (2) those who underwent ancillary vascular tests to assess peripheral vascular status using the ankle-brachial index and toe-brachial index; and (3) those who underwent an ophthalmic examination within 6 months after DFU diagnosis. A total of 161 consecutive patients were reviewed during the observation period from October 2004 to October 2011. Of the 161 patients reviewed, 19 patients were excluded from the study because they did not undergo a vascular laboratory test using the ankle-brachial index or toe-brachial index. Additionally, 42 patients were excluded from the study because they had not undergone an ophthalmic examination within 6 months after DFU diagnosis. Thus, finally, 100 patients were enrolled.

The prevalence and severity of DR were assessed for DFU patients who met the inclusion criteria. In addition, the association between the severities of DR and DFU were evaluated. Patients with DFUs were divided into two groups according to DR severity (proliferative DR vs. non-proliferative DR). Demographic, clinical, and biochemical characteristics were compared between the two groups. The management of hyperglycemia was categorized into four groups: no medication, an oral insulin sensitizer alone, combined oral agents, and insulin.

#### Control group of diabetic patients without DFU

The medical records of 2496 type 2 diabetic patients without a DFU who visited a healthcare center at Seoul National University Bundang hospital for a health checkup from July 2004 to June 2008 were reviewed. The medical checkups consisted of history taking for systemic diseases and health behaviors, for example, smoking, physical examinations by physicians, laboratory tests on peripheral blood and urine, and eye examinations. Systolic and diastolic blood pressures (BPs), height, and weight were measured and BMI ([weight in kg]/[height in m]^2^) was calculated. Laboratory tests included a complete blood cell count (CBC), hematocrit, white blood cell count (WBC), and levels of blood glucose, hemoglobin A1c (HbA1c), cholesterol, triglycerides, LDL, HDL, and serum creatinine.

### Grades of DR

Complete ophthalmologic examinations including funduscopy on the entire retina after mydriasis were performed on all patients with DFU by two retina specialists. After the thorough funduscopic examination, patients showing any features of diabetic retinopathy underwent color fundus photography using mydriatic 45° fundus camera (VX-10α, Kowa Inc., Nagoya, Japan). Subsequent fundus fluorescein angiography using the same fundus camera (VX-10α) was performed on patients who showed diabetic retinopathy more severe than mild non-proliferative diabetic retinopathy (NPDR). For the control group of diabetic patients without DFU, fundus photography on each eye was performed by an experienced technician using a nonmydriatic 45° fundus camera (Retinal Camera CR6-45NM; Canon Inc., Tokyo, Japan). A single fundus photograph centered on the fovea was taken for each eye. All retinal photographs of diabetic patients were independently reviewed twice by two ophthalmologists. When there was disagreement regarding the DR stage between the two ophthalmologists, the photograph was reviewed again by the two ophthalmologists and finally graded after discussion. The presence and severity of DR were graded based on international clinical DR severity scales proposed by the Global Diabetic Retinopathy Project Group [11]. The five-stage disease severity classification of DR consists of no apparent retinopathy (no DR); mild, moderate, or severe non-proliferative DR (NPDR); and proliferative DR (PDR). NPDR was defined as the presence of at least one definite retinal hemorrhage and/or microaneurysm. Subjects were assigned to PDR when retinal neovascularization was visible on retinal photographs or fundus fluorescein angiography. When two eyes in one patient had different diabetic retinopathy severity stages, the more severe stage was allocated to the subject.

### Grades of DFUs

Patients were grouped according to the Wagner ulcer classification system[12] by orthopedic staff (P.M.S.) to classify foot lesions as follows: grade 0 (no skin lesion with hyperkeratosis below or above bony prominences); grade 1 (ulceration of the skin and immediate subcutaneous tissue); grade 2 (deeper lesions that may penetrate to the tendon, bone, or joint capsule); grade 3 (deep tissues are always involved, and osteomyelitis may be present); grade 4 (gangrene of some portion of the toes or forefoot); and grade 5 (the entire foot is gangrenous) [13]. This study excluded patients with grade 0 feet, who had no ulcerative lesion on their skin.

### Ankle-brachial index/toe-brachial index

In patients with DFUs, the ABI and TBI were measured using a VasoGuard device (Nicolet Vascular, USA) and a Flo-Lab 2100SX device (Parks, USA) that allows for simultaneous systolic BP measurements from both the upper and lower extremities. To obtain the most accurate results, physical activity was limited during the 3-hour period before the evaluations. The BPs of the lower extremities were measured after patients had been lying still on a table for about 15 minutes. The ABI was calculated by measuring the systolic pressure in the brachial artery and in both the dorsalis pedis and posterior tibial arteries [14, 15], and the systolic pressures of each leg were divided by the brachial pressure. Similarly, the TBI was calculated by dividing the systolic pressure of the great toe by that of the brachial artery [14, 16]. Probes were attached to the tips of both great toes, and cuffs were placed on the arms and legs above the ankle or at the base of the great toe.

### Ethics statement

The study was approved by the institutional review board (IRB) of Seoul National University Bundang Hospital (SNUBH) and the SNUBH IRB waived the need for the patient consent. The study was carried out in accordance with the tenets of the Declaration of Helsinki.

### Statistical analyses

Statistical analyses were performed using a commercially available software package (IBM SPSS Statistics 18; SPSS Inc., Chicago, IL, USA). Univariable analyses were performed on demographic data, clinical data, and biochemical characteristics, and the mean values and frequencies were compared between 1) the DFU and control (diabetes without DFU) groups, and 2) the DFU with PDR and DFU without PDR groups. Significant differences between the two groups were evaluated using the independent t test and Mann-Whitney U test for parametric and nonparametric data, respectively. Significant variables with P < 0.1 in the univariable analyses were used in the multivariable logistic regression analysis to show independent associations. In addition, the relationship between DR and DFU severities based on the Wagner ulcer classification were evaluated by Pearson’s correlation analysis. The severity of DR and the ABI or TBI value were also analyzed using the independent t test. Statistical significance was defined as P < 0.05.

## Results

### Baseline characteristics of patients with a DFU

**Fig 1:**
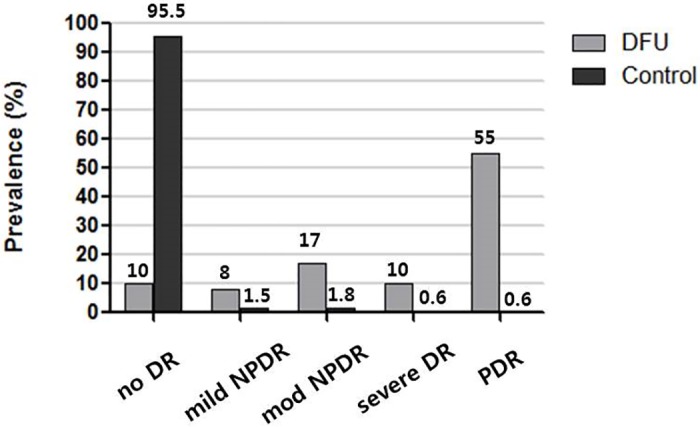
Prevalence of diabetic retinopathy (DR) in patients with a diabetic foot ulcer (DFU) and in control diabetic patients without a DFU. Among patients with a DFU, 90 (90%) had DR and 55 (55%) had proliferative DR (PDR). However, only 16 (0.6%) of 2496 control group patients without a DFU had PDR.

The mean age of patients with a DFU was 66.7 ± 10.6 years (range, 35–90 years), and 74 patients (74%) were men. The mean duration of diabetes mellitus (DM) was 18.5 ± 10.6 years and the HbA1c level averaged 8.0 ± 1.8% (64 ± 19.7mmol/mol). Table 1 shows the demographic, clinical, and laboratory characteristics of the subjects with DFUs. Among 100 patients with DFUs, only one patient (1%) had a grade 5 ulcer. Most patients (35%) had a grade 1 ulcer. Ten patients (10%) had grade 2 ulceration, 26 patients (26%) had grade 3 ulceration, and 28 patients (28%) had grade 4 ulceration. In terms of DR, 90 patients (90%) had DR and 55 patients (55%) had proliferative diabetic retinopathy (PDR). Eight patients (8%) had mild non-proliferative diabetic retinopathy (NPDR) and 17 patients had moderate NPDR (17%). Severe NPDR was observed in 10 patients (10%), as shown in Fig 1.

**Table 1:** Demographic, clinical, and laboratory characteristics of 100 type 2 diabetic patients with a diabetic foot ulcer (DFU) and 2496 type 2 diabetic patients without a DFU.

	Diabetes		p value	DFU		p value
	N = 2596			N = 100		
	Diabetes with DFU	Diabetes Without DFU		DFU with PDR	DFUwithout PDR	
	N = 100	N = 2496		N = 55	N = 45	
**Demographic characteristics**						
Age, years	66.7±10.6	55.5±10.2	<0.001	66.7±8.8	66.8±12.6	0.940
Male gender	74 (74%)	1789 (72%)	0.583	41 (75%)	33 (73%)	0.891
Diabetes duration (years)	18.5±10.6	-	-	20.6±10.4	15.8±10.3	0.022
History of HTN	72 (72%)	486 (19%)	<0.001	41 (75%)	31 (69%)	0.531
Blood pressure (mmHg)						
Systolic BP	130.6±19.6	123.7±15.9	0.001	132.0±19.6	128.8±19.5	0.420
Diastolic BP	69.4±10.6	76.5±11.0	<0.001	69.4±9.4	69.3±12.0	0.968
Pulse pressure	60.0±17.6	47.1±12.5	<0.001			
History of Smoking	38 (38%)	1152 (46%)	0.110	18 (33%)	20 (44%)	0.230
BMI (kg/m^2^)	23.4±3.8	25.1±3.1	<0.001	23.5±3.9	23.2±3.6	0.659
**Biochemical characteristics**						
HbA1c (%, mmol/mol)	8.0±1.8, 64±19.7	7.4±1.3, 57±14.2	0.003	8.0±1.9, 64±20.8	8.0±1.8, 64±19.7	0.979
Preprandial glucose (mg/dL)	159.9±72.6	145.9±40.4	0.058	150.0±85.3	141.1±93.8	0.666
C-peptide	2.1±1.7	-	-	2.4±2.1	1.7±1.2	0.107
Insulin	26.2±27.9	-	-	18.8±13.0	34.0±36.7	0.103
Cholesterol (mg/dL)	150.7±38.6	202.6±41.0	<0.001	150.4±41.5	151.1±35.3	0.927
Triglyceride^a^ (mg/dL)	132.8±82.5	161.8±98.9	0.001	138.6±95.8	126.1±63.9	0.616
HDL (mg/dL)	42.1±13.1	52.6±12.7	<0.001	40.5±11.0	44.0±15.2	0.224
LDL (mg/dL)	82.5±33.6	106.9±29.4	<0.001	82.5±33.6	82.5±33.6	0.372
Hematocrit (%)	35.0±6.5	44.5±4.0	<0.001	33.9±6.9	36.4±5.6	0.055
BUN (mg/dL)	28.7±17.3	-	-	32.8±18.4	23.7±14.4	0.008
Creatinine (mg/dL)	3.0±3.2	1.1±0.3	<0.001	4.1±3.8	1.7±1.6	<0.001
ABI	0.96±0.34	-	-	0.96 0.38	0.96 0.29	0.983
TBI	0.59±0.29	-	-	0.58 0.29	0.61 0.30	0.722
DM foot ulcer						0.671
Gr1	35 (35%)	-	-	18 (33%)	17 (38%)	
Gr2	10 (10%)	-	-	4 (7%)	6 (13%)	
Gr3	26 (26%)	-	-	15 (27%)	11 (24%)	
Gr4	28 (28%)	-	-	17 (31%)	11 (24%)	
Gr5	1 (1%)	-	-	1 (2%)	0 (0%)	
DR			<0.001			<0.001
no DR	10 (10%)	2384 (95.5%)		0 (0%)	10 (22%)	
mild NPDR	8 (8%)	37 (1.5%)		0 (0%)	8 (18%)	
moderate NPDR	17 (17%)	44 (1.8%)		0 (0%)	17 (38%)	
severe NPDR	10 (10%)	15 (0.6%)		0 (0%)	10 (22%)	
PDR	55 (55%)	16 (0.6%)		55 (100%)	0 (0%)	
Any DR	90 (90%)	112 (4.5%)	<0.001			
**Methods of glycemic control**						
No medication	6 (6%)	598 (24%)	<0.001	6 (10%)	0 (0%)	0.070
Medication	94 (94%)	1898 (76%)		49 (90%)	45 (100%)	
Insulin sensitizer only	0 (0%)	-		0 (0%)	0 (0%)	
Combined oral agents	35 (35%)	-		19 (35%)	16 (36%)	
Insulin	59 (59%)	-		30 (55%)	29 (64%)	

^a^ For triglyceride, nonparametric test results were presented.

Continuous values are expressed as the mean ± SD; p values < 0.05 are indicated in a bold font. DFU = diabetic foot ulcer; DR = diabetic retinopathy; PDR = proliferative diabetic retinopathy; pulse pressure = systolic blood pressure—diastolic blood pressure; BMI = ([weight in kilograms]/[height in meters]^2^)

Patients with DFU were compared with the control (diabetes) group (N = 2496) in terms of each clinical variable. The DFU group was older in age, and demonstrated higher pulse pressure (systolic BP—diastolic BP), lower BMI, lower cholesterol and hematocrit levels, and higher HbA1c and serum creatinine levels than the control group, as shown in Table 1. Additionally, patients with DFUs were divided into two groups according to DR severity, and demographic, clinical, and biochemical characteristics were compared between the two groups. The PDR group had a longer duration of diabetic mellitus (20.6 vs. 15.8 years), and higher BUN (32.8 vs. 23.7 mg/dL) and serum creatinine (4.1 vs. 1.7 mg/dL) levels than the NPDR group.

### A comparison of DFU versus non-DFU patients using multivariable logistic regression analysis

Patients with DFU and diabetic patients without DFU were compared. The DFU group had a higher prevalence of PDR (OR, 306.27; 95% CI, 64.35–1457.80; p < 0.001) and DR (OR, 226.12; 95% CI, 58.07–880.49; p < 0.001), higher HbA1c level (OR, 1.97; 95% CI, 1.46–2.67; p < 0.001), higher serum creatinine level (OR, 1.62; 95% CI, 1.06–2.50; p = 0.027), lower hematocrit level (OR, 0.80; 95% CI, 0.74–0.87; p < 0.001), older age (OR, 1.12; 95% CI, 1.06–1.17; p < 0.001), lower cholesterol level (OR, 0.94; 95% CI, 0.92–0.97; p < 0.001), lower BMI (kg/m^2^, OR, 0.87, 95% CI, 0.75–1.00; p = 0.044) and higher pulse pressure (OR, 1.03; 95% CI, 1.00–1.06; p = 0.025) than the control group, as shown in Table 2. Additionally, the PDR group with DFU was compared with the non-PDR group with DFU after adjusting for age, sex, duration of diabetic mellitus, BUN, and hematocrit levels. Serum creatinine level remained significantly associated with PDR (OR, 1.37; 95% CI, 1.05–1.78; p = 0.021), as shown in Table 2.

**Table 2:** Multivariable logistic regression analysis for comparisons 1) between the DFU group (n = 100) and control group (diabetes without DFU; n = 2496), and 2) between the DFU with PDR group (n = 55) and DFU without PDR group (n = 45).

	P value	OR	95% CI for OR	
			lower	upper
**DFU group vs control group**				
Sex^a^	0.212	1.86	0.70	4.93
Age	<0.001	1.12	1.06	1.17
BMI (kg/m^2^)	0.044	0.87	0.75	1.00
Pulse pressure	0.025	1.03	1.00	1.06
HbA1c (%)	<0.001	1.97	1.46	2.67
Glucose (mg/dL)	0.903	1.00	0.99	1.01
Cholesterol (mg/dL)	<0.001	0.94	0.92	0.97
Triglyceride (mg/dL)	0.448	1.00	1.00	1.01
HDL (mg/dL)	0.128	0.97	0.93	1.01
LDL (mg/dL)	0.008	1.05	1.01	1.08
Hematocrit (%)	<0.001	0.80	0.74	0.87
Creatinine (mg/dL)	0.027	1.62	1.06	2.50
PDR	<0.001	306.27	64.35	1457.80
DR^c^	<0.001	226.12	58.07	880.49
**DFU with PDR vs DFU without PDR**				
Sex^b^	0.918	0.95	0.34	2.64
Age	0.390	0.98	0.94	1.03
Diabetes duration (years)	0.065	1.05	1.00	1.10
Hematocrit (%)	0.545	0.98	0.91	1.05
BUN (mg/dL)	0.956	1.00	0.97	1.04
Creatinine (mg/dL)	0.021	1.37	1.05	1.78

^a^ The female sex was set as the reference category.

^b^ The absence of PDR was set as the reference category.

^c^ The absence of DR was set as the reference category. The odds ratios of PDR and DR were calculated by a separate model that included either DR or PDR with other clinical variables. As the odds ratios of PDR and DR were extremely large compared to those of other variables, they were omitted in the forest plot.

### Vascular LAB- ankle-brachial index/toe-brachial index

**Fig 2:**
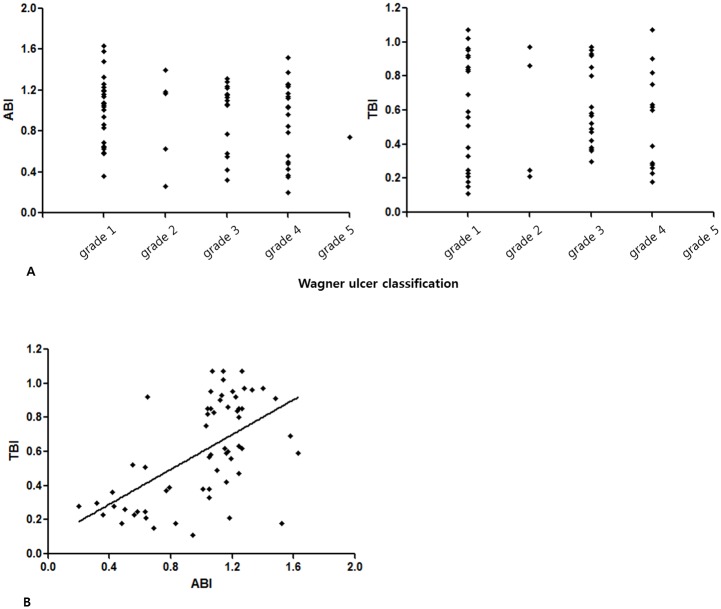
The associations between the Wagner ulcer classification system, ankle-brachial index (ABI), and toe-brachial index (TBI). (A) The Wagner ulcer classification system and ABI/TBI showed no correlation (p = 0.178 and 0.295, respectively). (B) The ABI and TBI showed a significant correlation (R = 0.573, p < 0.001).

In patients with DFU, the mean ABI value was 0.96 ± 0.34 and the mean TBI value was 0.59 ± 0.29. The severity of DFU by the Wagner ulcer classification system showed no significant correlation with the ABI or TBI (P = 0.178 and 0.295, respectively). However, the ABI and TBI showed a significant correlation (R = 0.573, p < 0.001) (Fig 2).

### Correlation between the severities of DR and DFU

**Fig 3:**
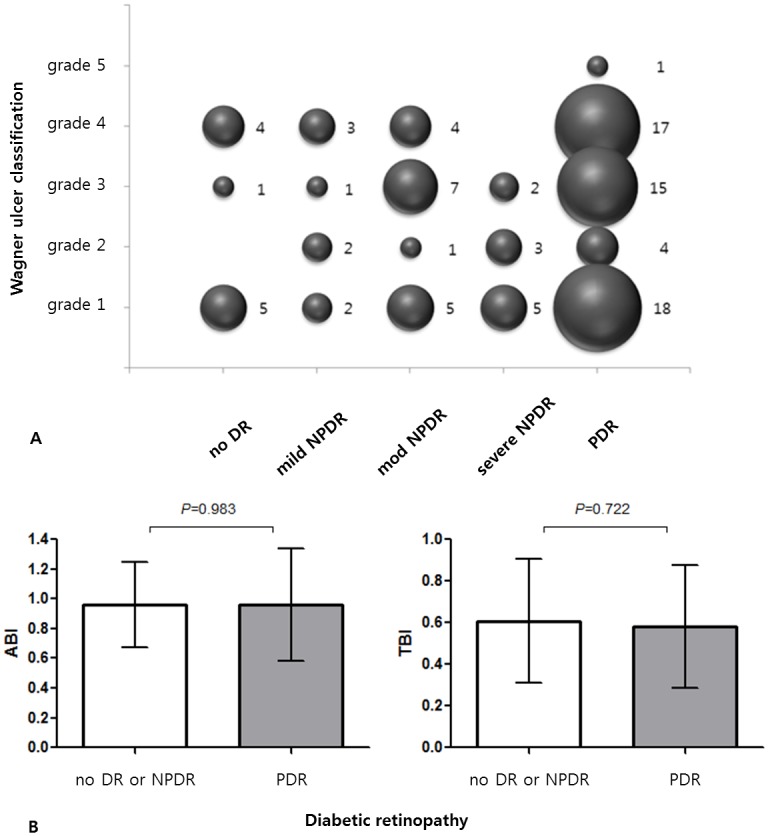
Correlations between the severities of diabetic retinopathy (DR) and diabetic foot ulcer (DFU) or other vascular index. (A) No correlation was shown between the severities of DR and DFU (*R* = 0.034, p = 0.734, Pearson’s correlation analysis). The numbers adjacent to the circles indicate sample size. (B) The severity of DR and ABI or TBI value also showed no significant association (p = 0.983 and 0.722, respectively, independent *t* test).

Pearson’s correlation analysis did not reveal a significant association between the severities of DR and DFU based on the Wagner ulcer classification, as shown in Fig 3 (R = 0.034, p = 0.734). The severity of DR and the ABI or TBI value also demonstrated no significant association (p = 0.983 and p = 0.722, respectively), as shown in Fig 3.

The results of the present study are summarized in Table 3.

**Table 3:** Summary of the results of the present study.

Data sources	Comparison	N	Statistical analysis	Corrections	Significant Findings (Odds Ratio)	DetailedResults
Diabetic patients	DFU vs non-DFU	2596 (100 vs 2496)	Univariable	-	*DFU patients*: Prevalence of DR/PDR↑, HbA1c↑, Cr↑, Age↑, Pulse pressure↑, Cholesterol↓, BMI↓, hematocrit↓	Table 1
			Multivariable Logistic regression	Age, Sex, BMI, Pulse pressure, HbA1c, Glucose, Cholesterol, TG, HDL, LDL, Hct, Cr, Presence of PDR,	*DFU patients*: Prevalence of DR↑ (226.12)/ PDR↑ (306.27), HbA1c↑ (1.97), Cr↑ (1.62), Age↑(1.12), pulse pressure↑ (1.03), cholesterol↓ (0.94), BMI↓ (0.87), hematocrit↓ (0.80)	Table 2
DFU patients	DFU with PDR vs DFU without PDR	100 (45 vs 55)	Univariable	-	*DFU with PDR*: Cr↑, BUN↑, DM duration↑	Table 1
			Multivariable Logistic regression	Age, Sex, DM duration, Hct, BUN, Cr	*DFU with PDR*: Cr↑ (1.37)	Table 2

DFU = diabetic foot ulcer; DR = diabetic retinopathy; Cr = creatinine; pulse pressure = systolic blood pressure—diastolic blood pressure; BMI = ([weight in kilograms]/[height in meters]^2^); Hct = hematocrit

## Discussion

The present study showed that the majority (90%) of patients with DFU also had DR, with more than half demonstrating PDR. When comparing diabetic patients with DFU with those without DFU who attended for health checkups, we found that age, increased pulse pressure, low cholesterol and hematocrit levels, and high HbA1c and serum creatinine levels were associated with an increased risk of developing a DFU. When classifying patients with a DFU into the PDR or NPDR group, there was an association between high serum creatinine levels and the presence of PDR. On the other hand, there was no significant correlation between DR severity and DFU severity.

Our study revealed that the prevalence of DR among diabetic patients without DFU was 4.5%, whereas it was 90% among those with a DFU. Moreover, PDR was present in 55% of patients with a DFU. A few studies have investigated the prevalence of DR in patients with DFU. Shahbazian et al.[17] showed that 33.3% of patients with a DFU of grade 1 or higher had DR. We speculate that the reason for the markedly lower prevalence of DR in their study than in ours was due to the younger age (66.7 vs 56.4 years) and shorter durations of diabetes (18.5 vs 9.83 years) in their cohort. The differences in the prevalence in DR between the two studies could be caused in part by differences in DFU severity, as our study included patients with higher DFU grades (grades 4 and 5). Jiang et al.[18] and Liu et al.[19] reported the prevalence of DR as 40.9% and 21.2%, respectively, in patients with DFU; however, both studies involved younger patients with shorter diabetes durations than in our study. Another difference was that in these two studies, serum creatinine levels were 0.91 and 0.76 mg/dL, respectively, which were lower than that in our study (3.0 mg/dL). We believe that the high prevalence of DR and PDR in our study compared to that in previous studies might be caused by the inclusion of patients with higher DFU grades who were admitted to a tertiary hospital in our study. Although we failed to show a correlation between higher DFU grade and DR severity (Fig 3), the prevalence of DR was shown to be associated with the presence of a DFU (Table 1). Thus, the inclusion of higher DFU grades in our study might have resulted in a higher prevalence of DR.

The fact that the majority (90%) of patients with a DFU had DR, including 55% with PDR, raises concerns about the impact of this combined disability on patients’ quality of life (QOL). There have been several reports of DFUs decreasing QOL [20, 21]. Specifically, increased rates of depression because of a DFU [22] and fear of amputation [23] lead to a lower QOL, as well as difficulty in controlling blood sugar, causing concerns about a high incidence of late complications of diabetes. Moreover, psychiatric problems and changes in lifestyle resulting from disability may place unexpected burdens on patients and their families. For example, about 50% of patients with DFUs are unable to work, and the remaining half have been reported to show decreased productivity with limitations in career advancement [24]. Moreover, DR has also been reported to decrease QOL [25–27]. The progression of DR into PDR usually causes significant and disabling vision loss, which leads to an even more significant decrease in QOL [28]. Therefore, from a QOL perspective, patients with both a DFU and DR, and particularly those with PDR, are in a very serious condition. We believe that it is essential to refer patients to ophthalmologists when they are diagnosed with a DFU. To prevent any further decrease in QOL, the timely diagnosis and treatment of DR is crucial.

Our study demonstrated that age, increased pulse pressure, increased HbA1c and serum creatinine levels, and low cholesterol, BMI and hematocrit levels increased the risk of developing a DFU. There have been previous studies on risk factors for DFU; Shahbazian et al.[17] also reported advanced age and higher HbA1c levels as risk factors. Monami et al.[29] elucidated that elevated pulse pressure was an independent risk factor for developing a DFU. Boyko et al.[30] also reported HbA1c and serum creatinine levels as risk factors for developing a DFU. Hence, the results of our study were consistent with those of previous reports. Compared to the control patients, the DFU patients in this study were older; had higher pulse pressure, HbA1c, and Cr values; and had lower cholesterol and hematocrit values. Even though the duration of DM for the control group has not been provided, the mean duration of DM for the DFU patients was 18.5 years, which is a long period of time, as compared to symptomless DM patients who simply visit the hospital for a health check-up. Therefore, we believe that DFU patients represent patients with several comorbidities, including capillary diseases of the kidneys and retina. The mean Cr of the DFU patients was 3.0, suggesting that the majority of this group have chronic renal failure. Based on a DM history and health measures of the DFU patients, it is likely that chronic renal failure in these patients progressed to the anemia of chronic disease, that arteriosclerosis and stiffness of the arteries caused widening of the blood pressure, and that malnutrition led to a decline in cholesterol and BMI.

Although risk factors for PDR among diabetic patients have been well described in previous reports, there has been no report investigating risk factors for PDR solely among patients with DFU. In addition, our study showed that impaired renal function due to an increase in serum creatinine had a strong association with PDR in patients with DFUs. Thus, DFU patients with high serum creatinine levels should undergo a retinal examination as soon as possible.

We studied the correlation between the severities of DFU and DR; however, no significant correlation was found. As there was no previous report studying the correlation between these two diseases, we were unable to compare our findings with previous studies. This might be due to differences in the pathological mechanisms underlying DR and DFU. Microvascular angiopathy is the main mechanism of DR. However, multiple mechanisms seem to contribute to the development of a DFU, such as neuropathy, macrovascular angiopathy, microangiopathy, and infection [31]. Neuropathy is a major factor contributing to DFU, with motor nerve involvement causing foot deformities and causing the foot to be more sensitive to pressure and more susceptible to wounding. Since sensory nerve dysfunction causes the foot to be numb, it is an important predisposing factor for pressure ulcers. Autonomic nerve dysfunction causes impaired sweating, leading to dry, flaking skin and greater susceptibility to injury. In addition, because the foot is regularly subjected to pressure, many additional factors may contribute to the formation of a DFU, such as activity levels and poorly fitted footwear. Furthermore, unlike with DR, infection occurs relatively frequently with DFUs, which is another factor distinct to DFUs that makes it difficult to explain DR and DFU with a single pathogenesis. Impaired healing ability, impaired regenerative abilities, and infection are thought to be the reasons that we did not observe a correlation between DFU grade and DR stage. However, our study also showed that the prevalence of DR and PDR was strongly associated with DFU, which suggests a shared pathogenic mechanism between DR and DFU. DR and DFU share a common pathogenic mechanism as vascular disease, which is characterized by oxidative stress and endothelial dysfunction. We suspect this is the reason for the higher PDR prevalence in DFU patients.

The lack of an association between ABI, TBI, and DFU severity can be understood along these lines as well. DR reflects the microvascular state of the disease, whereas ABI and TBI are values that reflect the macrovascular state [15], which did not correlate with DFU severity. In patients with a DFU, vascular calcification is frequently observed, and the ABI and TBI are often overestimated. Therefore, ABI and TBI are occasionally unreliable methods for evaluating lower extremity perfusion. This indicates that a DFU cannot be explained merely based on the macrovascular state, and that multiple factors are involved in the severity of a DFU.

Our study has several limitations, primarily due to its retrospective nature. First, because of the small number of DFU patients, the statistical power was limited to reveal the complete set of clinical factors associated with DR in patients with DFU and to confirm a non-significant association between DR and DFU severities. Therefore, prospective, well-designed studies are required to confirm and address these issues. Second, the diabetic control group was a large group of Type 2 diabetes patients who came to the clinic for a health checkup and was not selected to be matched with DFU group. Our control group patients can represent the general type 2 diabetic patients without DFU in Korea. However, the use of a healthier control patient group might have yielded an exaggerated result, although we tried to statistically adjust the unmatched clinical factors using multivariable logistic regression analysis. This might have caused a selection bias. In addition, the DR grading of diabetic patients without DFU using fundus photography was different from the DFU who received thorough ophthalmic examinations. This might have caused under-estimation of DR/PDR in control diabetic patients without DFU and might have exaggerated the odds ratio. However, as we performed a multivariable analysis adjusting related factors including HbA1c levels, and as the number of control diabetic patients was large, the selection bias might not have affected the result significantly. Third, our study was a cross-sectional in nature. Thus, the long-term consequences of DR and DFU are unknown. Future studies are needed to investigate whether the progression of DR correlates with the development or worsening of a DFU.

In conclusion, most patients with DFU have DR, and more than half of the patients had the sight-threatening condition of PDR, although the severities of these two diabetic complications had no significant association. Therefore, patients with DFUs, and particularly those with high serum creatinine levels, should undergo an immediate retinal examination for the timely diagnosis and treatment of DR.

## Supporting information

S1 File Dataset. (XLSX)
